# Maternal and Infant Lipid-Based Nutritional Supplementation Increases Height of Ghanaian Children at 4–6 Years Only if the Mother Was Not Overweight Before Conception

**DOI:** 10.1093/jn/nxz005

**Published:** 2019-04-29

**Authors:** Sika M Kumordzie, Seth Adu-Afarwuah, Mary Arimond, Rebecca R Young, Theodosia Adom, Rose Boatin, Maku E Ocansey, Harriet Okronipa, Elizabeth L Prado, Brietta M Oaks, Kathryn G Dewey

**Affiliations:** 1 Program in International and Community Nutrition, Department of Nutrition, University of California, Davis, CA; 2 Department of Nutrition and Food Science, University of Ghana, Ghana; 3 *Intake* – Center for Dietary Assessment, FHI 360, Washington, DC; 4 Nutrition Research Centre, Radiological and Medical Sciences Research Institute, Ghana Atomic Energy Commission, Legon, Ghana; 5 Department of Nutrition and Food Sciences, University of Rhode Island, Kingston, RI

**Keywords:** growth, body composition, lipid-based nutrient supplements, follow-up, prenatal supplementation

## Abstract

**Background:**

Few studies have evaluated the long-term effects of nutritional supplementation during the first 1000 d of life. We previously reported that maternal and child lipid-based nutrient supplements (LNS) increased child length by 18 mo.

**Objective:**

The aim of this study was to examine the effects of LNS on later growth and body composition at 4–6 y of age.

**Design:**

This was a follow-up of children in the International Lipid-based Nutrient Supplements (iLiNS)-DYAD trial in Ghana. Women (*n* = 1320) at ≤20 weeks of gestation were randomly assigned to: *1*) iron and folic acid during pregnancy and 200 mg calcium/d for 6 mo postpartum, *2*) multiple micronutrients (1–2 RDA of 18 vitamins and minerals) during both periods, or *3*) maternal LNS during both periods plus child LNS from 6 to 18 mo. At 4–6 y, we compared height, height-for-age *z* score (HAZ), and % body fat (deuterium dilution method) between the LNS group and the 2 non-LNS groups combined.

**Results:**

Data were available for 961 children (76.5% of live births). There were no significant differences between LNS compared with non-LNS groups in height [106.7 compared with 106.3 cm (mean difference, MD, 0.36; *P* = 0.226)], HAZ [−0.49 compared with −0.57 (MD = 0.08; *P* = 0.226)], stunting (< -2 SD) [6.5 compared with 6.3% (OR = 1.00; *P* = 0.993)], or % body fat [15.5 compared with 15.3% (MD = 0.16; *P* = 0.630)]. However, there was an interaction with maternal prepregnancy BMI (kg/m^2^) (*P*-interaction = 0.046 before correction for multiple testing): among children of women with BMI < 25 , LNS children were taller than non-LNS children (+1.1 cm, *P* = 0.017), whereas there was no difference among children of women with BMI ≥ 25 (+0.1 cm; *P* = 0.874).

**Conclusions:**

There was no overall effect of LNS on height at 4–6 y in this cohort, which had a low stunting rate, but height was greater in the LNS group among children of nonoverweight/obese women. There was no adverse impact of LNS on body composition. This trial was registered at clinicaltrials.gov as NCT00970866.

## Introduction

Nutrition is essential for child growth and development throughout the first 1000 d of life (conception to the child's second birthday) and beyond (1–3). Early childhood nutrition has been linked to several health outcomes in later life including obesity and chronic diseases such as type 2 diabetes, hypertension, and cardiovascular disease (4). The relationships of child anthropometric indices to later health may depend on the index examined. Stunting is a risk factor for diminished survival, learning capacity, and productivity (2, 5). On the other hand, rapid increases in weight and BMI during the preschool years are associated with adult obesity and altered body composition in adolescence and adulthood (6).

Although several randomized trials have examined the effects of nutritional supplementation in early life, very few have included follow-up of long-term consequences. Two landmark trials of maternal plus child food supplementation conducted in the 1970s included assessments several years post intervention. In Guatemala (7), pregnant and lactating women and their children from birth to 7 y of age received either a high-protein, high-energy supplement called “Atole” or a nonprotein, low-energy supplement called “Fresco,” both fortified with micronutrients. In adolescence, children in the Atole group were taller, heavier, and had higher fat-free mass (FFM) than those in the Fresco group. In Colombia, there was a sustained positive effect of maternal and child food supplementation on child height and weight 3 y after the intervention period ended (8).

The International Lipid-based Nutrient Supplements (iLiNS)-DYAD trial in Ghana is 1 of the first randomized trials since the 1970s to assess the impact of combined maternal and infant supplementation on child growth and other outcomes (9, 10). The iLiNS-DYAD trial tested the efficacy of small-quantity lipid-based nutrient supplements for both mothers and infants, compared with 2 other treatments for mothers only [prenatal iron and folic acid (IFA) followed by postnatal calcium (200 mg/d) supplementation, or multiple micronutrients (MMN) both prenatally and postnatally], with regard to pregnancy outcomes and child growth to 18 mo of age. At birth, infants born to women in the LNS group had significantly greater birth weight, weight-for-age, and BMI-for-age *z* scores compared to children born to women in the IFA group but not the MMN supplement group; among infants of primiparous women, the LNS group had greater weight and length compared to both control groups (10). By 18 mo, there was a significant positive effect of LNS on length, stunting prevalence, and weight compared to the 2 control groups combined (9). Our objective herein is to examine the effect of maternal and infant supplementation with LNS on child growth and body composition at 4–6 y of age in the iLiNS-DYAD Ghana cohort.

## Methods

### Location and study design of the main trial

The iLiNS-DYAD Ghana trial was conducted in the Yilo and Lower Manya Krobo districts of the Eastern Region of Ghana between December 2009 and March 2014. Details of the study are published elsewhere (10). Briefly, the iLiNS-DYAD study was a partially double-blind randomized controlled trial which enrolled 1320 women aged ≥18 y at ≤20 weeks of gestation attending antenatal clinics in 4 main health facilities in the study area. The women were randomized to 1 of 3 treatments: (1) daily IFA tablets during pregnancy and a 200 mg/d calcium tablet (placebo) during the first 6 mo postpartum and no infant supplementation, (2) daily MMN tablets (1–2 RDA of 18 vitamins and minerals) during pregnancy and the first 6 mo postpartum and no infant supplementation, or (3) daily 20 g (118 kcal) lipid-based nutrient supplements (LNS) during pregnancy and the first 6 mo postpartum followed by infant 20 g/d LNS supplementation from 6 to 18 mo of age. The maternal LNS had the same micronutrient content as the MMN, plus calcium, magnesium, phosphorus, potassium, and macronutrients (essential fatty acids and a small amount of protein), and the infant LNS had the same macronutrients and 22 micronutrients based on infant Recommended Nutrient Intakes (11). Data collection in the main trial ended for women at 6 mo postpartum and for the children at 18 mo of age.

### Follow-up when children were 4–6 y of age

After the main trial ended in 2014, an update of contact information was undertaken in preparation for a future follow-up study. The follow-up study occurred in 2016 when the children were 4–6 y of age.

#### Participants at follow-up

**FIGURE 1:**
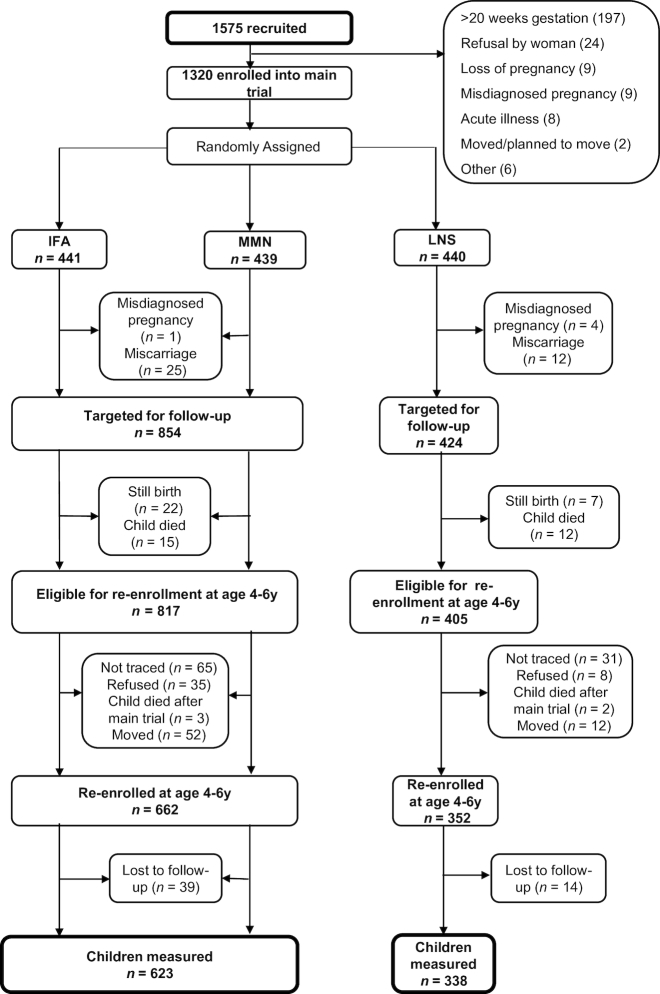
Study profile of the International Lipid-Based Nutrient Supplements (iLiNS)-DYAD Ghana trial. IFA, iron folic acid; LNS, lipid-based nutrient supplement; MMN, multiple micronutrients.

For the follow-up at 4–6 y of child's age, we sought to locate all children born to pregnant women who had been randomized to 1 of the 3 intervention groups of the iLiNS-DYAD trial, regardless of whether their mothers were lost to follow-up before delivery or they themselves or their mothers were lost to follow-up after delivery and before the end of the main trial. Excluding misdiagnosed pregnancies (*n* = 5), miscarriages, and stillbirths (*n* = 66), and children who died before the end of the main trial (*n* = 27), 1222 children were potentially eligible to participate. Details of participant enrollment and eligibility are shown in **Figure 1**.

The follow-up study protocols were approved by the Institutional Review Boards of the University of California, Davis, the Ethics Committee for the College of Basic and Applied Sciences at the University of Ghana, and the Ghana Health Service Ethical Review Committee. Both the main trial and follow-up were registered at clinicaltrials.gov as NCT00970866. Written informed consent was given by the primary caregiver before data collection.

#### Data collection procedures

Before data collection for the follow-up began, we conducted a pilot study using the deuterium dilution method to determine the equilibration time for a deuterium dose in 4–6-y-old children. The pilot study followed the procedures outlined by the International Atomic Energy Agency for determination of total body water (TBW) using a Fourier transform infrared spectrometer (12). The sample size for the pilot study was 15 children. The children consumed a dose of deuterium based on their body weight. No adverse effects of deuterium oxide at this amount have been reported in humans (12). Saliva samples were collected at baseline before the dose, 2 h after the dose, and every 30 min until 4 h after the dose was given. Data were analyzed by plotting a graph of mean deuterium concentration against time of post-dose sample collection, which showed a plateau between 2.5 h and 3.5 h. To determine the equilibration time, we calculated each child's % CV for deuterium enrichment for each pair of time points, between 2 h and 2.5 h, 2.5 h and 3 h, 3 h and 3.5 h, and 3.5 h and 4 h. Equilibration is considered to have occurred when the % CV is ≤2% (12). The mean % CV was 1.46, 0.61, 0.74, and 1.77 for the 2 h and 2.5 h, 2.5 h and 3 h, 3 h and 3.5 h, and 3.5 h and 4 h time points, respectively. Based on this analysis, we chose 2.5 h and 3 h after dose administration as the equilibration times to be used in the follow-up study.

For the follow-up, data collection was done at a central location. We used Open Data Kit software and programmed our questionnaires on tablets (13) with quality checks built into the system to minimize entry of implausible values. A questionnaire was administered to update information provided at enrollment into the main trial. Anthropometric measurements were taken by 2 anthropometrists who had been trained to take similar measurements in the main trial, 1 of whom was considered the “gold standard” for standardization sessions. The anthropometrists were retrained and standardized at the beginning of data collection and every quarter until the end of data collection (14).

Height was measured using a stadiometer (Seca 217) to 0.1 cm, weight to the nearest 50 g (Seca 875 scale), midupper arm circumference (MUAC) to the nearest 0.1 cm using a Shorr tape, and triceps skinfolds (TSF) to 0.2 mm with Lange calipers according to WHO standard procedures (15). All measurements were taken in duplicate and in triplicate if the first 2 measurements differed by a predefined amount: 0.1 kg for weight, 0.5 cm for height and MUAC, and 2 mm for TSF.

For TBW determination, children were given a dose of deuterium based on weight. Children <20 kg received a 6 g dose, whereas those ≥20 kg were given a 10 g dose as recommended by the International Atomic Energy Agency (12). All doses were prepared in bulk as a 1 in 5 dilution. If the child had been sick (defined as fever, cold, cough, malaria, hospitalization) in the previous 7 d before the day of data collection, the TBW assessment was rescheduled. Because the children were young, we required that all children be given breakfast before they came for sample collection, and the dose was administered at least 2 h after breakfast. A snack (150 g of a chocolate drink and a packet of biscuits) of <300 kcal was also provided 1 h after the dose to standardize what they were offered. Any leftovers were weighed and recorded. Saliva samples were stored in a −33°C freezer and transported in ice chests to the laboratory at the Ghana Atomic Energy Commission for analysis. Each sample was measured in duplicate and the mean deuterium concentration for each time point was calculated. Each morning and at different times during the sample analysis, performance of the equipment was checked by running quality control samples.

Percent CV was calculated using the means of the 2.5 h and 3 h time points. If the % CV was >5%, samples were rerun and if sample volume was not sufficient for the rerun or the % CV remained >5%, only the enrichment at 3 h was used in the calculation of TBW. For subjects without the 3 h sample, enrichment at 2.5 h was used in the calculation of TBW.

To determine TBW, the average of the means at 2.5 and 3 h was taken and used together with the amount of dose administered and dilution space of the body in the calculation of TBW. Where there was only a value for 1 time point, that value was used to calculate TBW. FFM was then calculated from TBW and age- and sex-specific hydration factors (12). The difference between body weight and FFM gives fat mass (FM).

Anthropometrists, field staff, and laboratory staff were blinded to the group assignments. The data analyst remained blinded until all decisions regarding outliers had been made.

#### Sample size and data analysis

We hypothesized that (a) children in the LNS group would differ in height and HAZ compared to children in the combined IFA and MMN tablet group at 4–6 y, and (b) children in the LNS group would not have significantly higher % body fat compared to the children in the combined IFA and MMN tablet (non-LNS) group at 4–6 y (noninferiority analysis). For the second hypothesis, we chose a noninferiority approach because our objective was to rule out potential adverse effects (i.e., greater body fatness in the LNS group), given concerns that a high-fat product might lead to greater body fatness, and we prespecified a noninferiority margin of 1% in body fat, based on a SD of 2.6% in fat mass (16) and the aim of ruling out any effect greater than a moderate effect size of 0.4 (noninferiority margin divided by SD). Noninferiority was established if the difference in the means and the CI around it were below the predetermined margin of inferiority (where lower is better). Both hypotheses were based on combining the 2 control groups (IFA and MMN), as there was no evidence of differences in growth or body composition in other studies comparing MMN to IFA (17). Nevertheless, a sensitivity analysis was also performed comparing the 3 groups to check for any differences between the IFA and MMN groups. Primary outcomes were height (cm), height-for-age *z* score (HAZ) and % FM at 4–6 y. Secondary outcomes included stunting (HAZ < -2), weight (kg), weight-for-age *z* score (WAZ), underweight (WAZ < -2), overweight (BMIZ > +1), arm fat area (AFA), arm muscle area (AMA), and high body fat. AFA and AMA were calculated using the following equations:

(1) \begin{equation*}
\begin{array}{@{}*{1}{l}@{}} {{\rm{AFA}}({\rm{c}}{{\rm{m}}^2}) = \text{(TSF X MUAC/2)} - {({\pi }\text{ X(TSF)}^{2}/4)}\\
{{\rm{AMA}}({\rm{c}}{{\rm{m}}^2}) = {{[({\rm{MUAC}} - ({\rm{\pi }}\,\,{\rm{X}}\,\,{\rm{TSF}}))]}^2}/4{\rm{\pi }}} \end{array}
\end{equation*}

where TSF and MUAC are in cm (18).

High body fat was defined as % body fat ≥20. About half the children in our sample were <5 y of age, and we could not find a reference for high body fat for children aged <5 y. There are documented sex differences in %FM after 5 y of age (19), but it is not clear whether a sex difference is generally found in children aged <5 y, or the magnitude of any such difference. Because of this uncertainty we were unable to use sex-specific cutoffs for %FM, and instead chose a somewhat arbitrary cutoff (20%) that was in between the 85th percentile reference values for boys and girls 5–6 y of age [85th percentile, 18.6% and 19.5% for 5-y-old and 6-y-old boys, and 21.5% and 23.0% for 5-y-old and 6-y-old girls, respectively] (19) and coincided with the 85th percentile in our sample.

The sample size for the primary outcome of height was based on detecting an effect size, Cohen's *d* (20), of ≥0.25 in mean height. This resulted in a minimum sample size of 198 per group to detect the difference with 80% power and α = 0.05. (16) Our goal, however, was to measure all of the children we could locate, which provided sufficient power to test our second hypothesis regarding % body fat, and to evaluate other secondary outcomes within this longitudinal cohort.

A statistical analysis plan was posted on the iLiNS Project website (www.ilins.org) before data analysis. Analysis was carried out based on a complete case intention-to-treat principle using SAS version 9.4. All tests except the noninferiority hypothesis were 2-sided at a 5% level of significance.

All continuous data were examined by univariate analysis to identify outliers. The TSF, AMA, and AFA variables were not normally distributed, therefore they were log-transformed.

ANCOVA was used to analyze the data testing the null hypothesis of no difference in means for continuous variables. All models included child age at follow-up. Fully adjusted models were also adjusted for a set of prespecified potential covariates. In addition to child age, the potential covariates included: sex of child, maternal gestational age at enrollment, nulliparity, maternal education and estimated prepregnancy BMI at enrollment (9), maternal height and asset score (21). The binary outcomes were analyzed using logistic regression first by running the unadjusted model, and then the adjusted model using the prespecified covariates. For all analyses, only covariates significantly associated with the outcome at 10% level of significance in a bivariate analysis were included in the final adjusted analysis, except for child age which was included in all models. We also tested for interaction with a set of prespecified covariates (maternal height, estimated prepregnancy BMI, nulliparity at enrollment, total years of schooling, and child sex). The test for interaction was considered significant at *P*-interaction <0.10. We conducted further analysis by stratifying the intervention groups (non-LNS compared with LNS) by categories of the significant effect modifier.

For the noninferiority hypothesis regarding % body fat, ANCOVA was used to analyze the data. The difference in the means of the 2 groups and associated 95% CI was compared with the predetermined margin of inferiority (1% in body fat).

## Results

Of the 1222 children who were eligible to participate in the follow-up, we enrolled 662 in the non-LNS group and 352 in the LNS group (Figure 1). By the end of the data collection period (January to December 2016), we were able to measure 79% of the eligible children, 623 in the non-LNS group and 338 in the LNS group. The reasons for nonparticipation in the follow-up were inability to trace the mother (*n* = 96), consent refusal (*n* = 43), child death after the main trial ended (*n* = 5), no longer residing in the study area (*n* = 64), and loss to follow-up after enrollment in the follow-up (*n* = 53). The proportion lost to follow-up was 23.7% in the non-LNS group and 16.5% in the LNS group (*P* = 0.005).

**Table 1** shows the background characteristics at enrollment into the main trial of women whose children were enrolled into the follow-up. More than 90% of the women were married or cohabiting, a third were nulliparous at baseline, and 10.9% were underweight (BMI < 18.5 kg/m^2^). Of the eligible children from the main trial, maternal baseline characteristics for those included in this follow-up (*n* = 961) were not significantly different from those of children who were not in the follow-up, except for nulliparity (32.2% among those included in the follow-up sample compared with 38.3% among those not included, *P* = 0.041, **Supplemental Table 1**). Among those included in the follow-up, there were no significant differences in background characteristics between the non-LNS and LNS groups (Table 1), except in household asset score, which was higher in the non-LNS group (*P* = 0.017). At follow-up, the mean age (mean ± SD) of the children was 4.9 ± 0.6 y and the percentage of boys in the sample was 48%, and these did not differ between groups.

**TABLE 1:** Background characteristics at enrollment into the main trial of women whose children were eligible for follow-up, and children in the International Lipid-Based Nutrient Supplements (iLiNS)-DYAD Ghana trial at follow-up^1^

Characteristic	Non-LNS (*n* = 623)	LNS (*n* = 338)
Maternal characteristics		
Age, y	26.8 ± 0.2	26.8 ± 0.3
Gestational age at enrollment, wk	16.1 ± 3.2	16.1 ± 3.3
Formal education, y	7.6 ± 3.4	7.6 ± 3.8
Married or cohabiting, %	93.7	92.6
Asset score^2^	0.07 ± 0.95	−0.09 ± 0.98^3^
Primiparous women, %	31.9	32.5
Weight, kg	61.7 ± 0.5	62.9 ± 0.7
Height, cm	158.9 ± 5.8	159.2 ± 5.4
Prepregnancy BMI^4, 5^, kg/m^2^	24.5 ± 4.7	24.9 ± 4.5
Overweight (BMI ≥ 25), %	30.4	34.1
Underweight (BMI < 18.5), %	13.1	6.9
MUAC^4^, cm	27.8 ± 4.2	28.3 ± 4.3
TSF^4^, mm	18.7 ± 7.4	19.4 ± 7.5
Child characteristics		
Age of child at follow-up, y	4.9 ± 0.6	5.0 ± 0.6
Sex of child, % boys	47.7	48.6

^1^Values are mean ± SD unless otherwise stated. LNS, lipid-based nutrient supplements group; MUAC, midupper arm circumference; non-LNS, iron folic acid group + multiple micronutrients group; TSF, triceps skinfold.

^2^Household asset score was constructed based on ownership of a set of assets (radio, television, refrigerator, and stove), lighting source, drinking water supply, sanitation facilities, and flooring materials, developed into an index (with a mean of 0 and SD of 1) using principal components analysis.

^3^Different from control, *P* < 0.05.

^4^Data available for less than the full sample. Sample size for these analyses of 611 compared with 334 (non-LNS compared with LNS) for weight, BMI, MUAC, and TSF because some of the women did not have baseline anthropometric data.

^5^Estimated prepregnancy BMI was calculated from estimated prepregnancy weight (based on polynomial regression with gestational age, gestational age squared, and gestational age cubed as predictors) and height at enrollment.

The means ± SD for height, HAZ, weight, WAZ, BMIZ, and %FM in this cohort were 106.5 ± 5.5 cm, −0.54 ± 0.95, 16.6 ± 2.2 kg, −0.71 ± 0.86, −0.56 ± 0.83, and 15.4 ± 4.9%, respectively. **Table 2** shows the continuous unadjusted anthropometric measures and body composition results. At 4–6 y, there were no significant differences between the children in the non-LNS compared with LNS groups for any of these outcomes. In the 3-group analysis, we also did not find any significant overall group differences or pairwise differences between the IFA and MMN groups (**Supplemental Table 2**); for the primary outcome, height, the mean ± SE values were 105.9 ± 0.3 cm, 106.6 ± 0.3 cm, 106.7 ± 0.2 cm for the IFA, MMN, and LNS groups, respectively (*P* = 0.075).

**TABLE 2:** Anthropometric and body composition measurements of children in the International Lipid-Based Nutrient Supplements (iLiNS)-DYAD Ghana trial at 4–6 y^1^

Variable	Non-LNS (*n* = 623)	LNS (*n* = 338)	*P*-value	Difference in mean (95% CI)	Ratio of the geometric mean (95% CI)
Height, cm	106.3 ± 0.2	106.7 ± 0.2	0.226	0.36 (−0.23, 0.95)	—
Height-for-age *z* score	−0.57 ± 0.04	−0.49 ± 0.05	0.226	0.08 (−0.05, 0.21)	—
Weight, kg	16.5 ± 0.1	16.7 ± 0.1	0.214	0.17 (−0.10, 0.43)	—
Weight-for-age *z* score	−0.74 ± 0.03	−0.67 ± 0.05	0.252	0.07 (−0.05, 0.18)	—
BMI-for-age *z* score	−0.58 ± 0.03	−0.55 ± 0.05	0.606	0.03 (−0.08, 0.14)	—
MUAC, cm	15.4 ± 0.1	15.5 ± 0.1	0.382	0.07 (−0.09, 0.23)	—
TSF^2^, mm	7.0 (6.9, 7.1)	7.1 (6.9, 7.2)	0.392	—	1.01 (0.98, 1.04)
AFA ^2^, cm^2^	5.4 (5.3, 5.5)	5.5 (5.3, 5.6)	0.399	—	1.01 (0.98, 1.05)
AMA ^2^, cm^2^	13.7 (13.6, 13.9)	13.7 (13.6, 14.0)	0.891	—	1.00 (0.98, 1.02)
FM ^3^, kg	2.6 ± 0.0	2.6 ± 0.1	0.491	0.05 (−0.08, 0.17)	—
% FM ^3, 4^	15.3 (15.0, 15.7)	15.5 (14.9, 16.0)	0.630	0.16 (−0.49, 0.81)	—
FFM ^3^, kg	14.0 ± 0.1	14.1 ± 0.1	0.389	0.10 (−0.13, 0.34)	—
% FFM ^3^	84.9 (84.4, 85.3)	84.6 (84.0, 85.2)	0.429	−0.29 (−1.02, 0.43)	—

^1^Values represent mean ± SE and the difference in mean (95% CI) unless otherwise stated. Results are based on ANCOVA (SAS PROC GLIMMIX). AFA, arm fat area; AMA, arm muscle area; FFM, fat-free mass; FM, fat mass; LNS, lipid-based nutrient supplements group; MUAC, midupper arm circumference; non-LNS, iron folic acid group + multiple micronutrients group; TSF, triceps skinfold.

^2^Values are geometric means (95% CI) and ratio of the geometric mean (95% CI). *n* = 622 compared with 337 (non-LNS compared with LNS).

^3^Values are based on less than the full sample for FM and %FM (599 compared with 326, non-LNS compared with LNS) and for FFM and %FFM (604 compared with 327, non-LNS compared with LNS) because some participants did not take part in the body composition procedure, there was insufficient sample for analysis, or there were data collection or lab issues.

^4^An outlier in the %FM variable was truncated using the value representing the 95th percentile of the distribution to make the distribution more normal.

The results for the noninferiority analysis indicate that children in the LNS group did not have higher % body fat compared with children in the non-LNS group: the upper end of the CI for the difference in means between the 2 groups (+0.81% body fat, Table 2) did not exceed our predetermined inferiority margin of 1%.

The binary anthropometric outcomes are presented in **Table 3**. Overall prevalence of stunting (6.4%) was low, few children were overweight (2.9%), and 16.1% had high body fat (≥20%). There were no significant differences between intervention groups in any of the binary outcomes in either the 2-group (Table 3) or 3-group (**Supplemental Table 3**) comparisons.

**TABLE 3:** Prevalence of underweight, stunting, overweight, and % body fat ≥20% among children in the International Lipid-Based Nutrient Supplements (iLiNS)-DYAD Ghana trial at 4–6 y^1^

	Non-LNS (*n* = 622)	LNS (*n* = 338)	OR (95% CI)	*P*-value
Stunting (HAZ < -2 SD)	—	—	—	
Prevalence, %	6.3	6.5	1.00 (0.58, 1.73)	0.993
Underweight (WAZ < -2SD)	—	—	—	
Prevalence, %	5.6	6.2	1.10 (0.63, 1.93)	0.730
Overweight (BMIZ > 1 SD)	—	—	—	
Prevalence, %	2.9	3.0	1.10 (0.47, 2.54)	0.827
Body fat ≥20%	—	—	—	
Prevalence ^2^, %	14.3	17.4	1.26 (0.87, 1.18)	0.218

^1^Values are the percentage of participants whose response was “yes” for the outcome in question and OR (95% CI) obtained by comparing the groups. Reference = non-LNS group for all outcomes. Results are based on logistic regression (SAS PROC GLIMMIX). BMIZ, BMI-for-age *z* score; HAZ, height-for-age *z* score; LNS, lipid-based nutrient supplements group; non-LNS, iron folic acid group + multiple micronutrients group; WAZ, weight-for-age *z* score.

^2^Values are based on less than the full sample for overweight (601 compared with 328, non-LNS compared with LNS) because some participants did not take part in the body composition procedure, there was insufficient sample for analysis, or there were data collection or lab issues.

Adjustment for prespecified covariates did not alter any of the above findings (data not shown).

**FIGURE 2:**
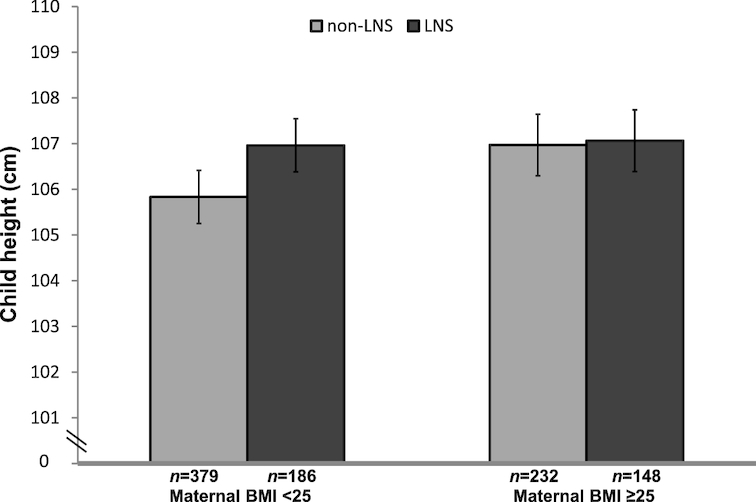
Child height at 4–6 y by intervention group (LNS compared with non-LNS), stratified by maternal BMI at enrollment into the International Lipid-Based Nutrient Supplements (iLiNS)-DYAD Ghana trial. Values represent mean (95% CI) from an ANCOVA model (SAS PROC GLIMMIX). *P*-interaction between estimated maternal prepregnancy BMI as a continuous variable and intervention group = 0.046, before Benjamini/Hochberg correction, adjusting for maternal height, years of education, nulliparity, asset score, and child sex. LNS, lipid-based nutrient supplements group; non-LNS, iron-folic acid group + multiple micronutrients group.

**FIGURE 3:**
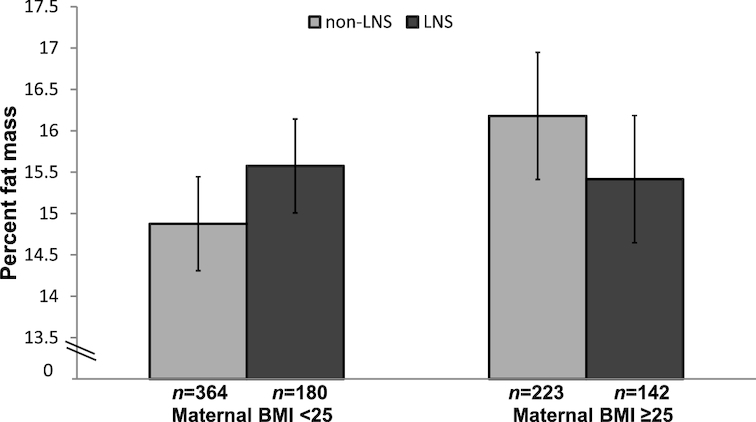
Child % fat mass at 4–6 y by intervention group (LNS compared with non-LNS), stratified by maternal BMI at enrollment into the International Lipid-Based Nutrient Supplements (iLiNS)-DYAD Ghana trial. Values represent mean (95% CI) from an ANCOVA model (SAS PROC GLIMMIX). *P*-interaction between estimated maternal prepregnancy BMI as a continuous variable and intervention group = 0.037, before Benjamini/Hochberg correction, adjusting for nulliparity and child sex. LNS, lipid-based nutrient supplements group; non-LNS, iron-folic acid group + multiple micronutrients group.

We examined potential effect modification by maternal height, BMI, primiparity, total years of schooling, and child sex. We found interactions (*P*-interaction <0.10, before correction using the Benjamini-Hochberg procedure) of intervention group with estimated maternal prepregnancy BMI for child height (**Figure 2**), HAZ, and % fat mass, and with maternal height for child % fat mass. For the interactions with maternal prepregnancy BMI, height was greater in the LNS group compared with the non-LNS group (+1.1 cm; 95% CI: 0.2, 2.1 cm, *P* = 0.017) among children whose mothers had a BMI < 25 kg/m^2^ (*n* = 565) at baseline, but there was no difference (+0.1 cm; 95% CI: −1.1, 1.3 cm, *P* = 0.874) among children of overweight mothers (BMI ≥ 25 kg/m^2^; *n* = 380) (Figure 2). A similar trend was observed for child HAZ: +0.19 (95% CI: 0.03, 0.35, *P* = 0.018) in the LNS group among children of mothers with BMI < 25 kg/m^2^, and no effect of LNS (−0.10; 95% CI −0.03, 0.11, *P* = 0.349) among children of overweight mothers. We did not observe a difference in % fat mass between the non-LNS and LNS groups when we stratified by maternal BMI (**Figure 3**), although *P*-interaction was 0.037. The interaction of intervention group with maternal height, with regard to child % fat mass (**Supplemental Figure 1**), was similar to the pattern shown with maternal BMI. However, none of the *P*-for-interaction values was significant after correction for multiple comparisons using the Benjamini/Hochberg procedure.

## Discussion

In this follow-up of the iLiNS-DYAD Ghana cohort, we did not observe any overall effect of LNS provided to mothers during pregnancy and the first 6 mo postpartum and to their children from 6 to 18 mo of age on child height, HAZ, or any of the other anthropometric measures at 4–6 y of age. Although there were significant differences in mean length (0.61 cm; *P* = 0.001) and weight (210 g; *P* = 0.010) between the LNS and non-LNS groups at 18 mo (9), these differences were not sustained several years later. However, in prespecified subgroup analyses, the results suggested that maternal BMI may have modified the effect of the intervention: among children of women who were not overweight at baseline, child height was +1.1 (95% CI: 0.2, 2.1) cm greater in the LNS group compared with the non-LNS group at 4–6 y, whereas there was no difference between intervention groups among children of overweight women.

The noninferiority results support our hypothesis that provision of LNS in early life did not increase % body fat at 4–6 y of age in the overall cohort. There was evidence suggesting effect modification by maternal prepregnancy BMI, with slightly greater % body fat in the LNS (compared with non-LNS) group among children of women who were not overweight at baseline, but slightly lower % body fat in the LNS (compared with non-LNS) group among children of overweight women. Because the difference between intervention groups was not significant in either of these subgroups, we cannot conclude that there were any positive or negative effects, although Figure 3 suggests that LNS eliminated the differential between children of nonoverweight and overweight mothers: % body fat in the LNS group was similar across both subgroups, whereas in the non-LNS group % body fat was 1.5% higher in the subgroup of children born to overweight mothers. The overall percentage of children with high body fat (≥20%) was 16.1%, which is not excessive given that the cutoff was based on the 85th percentile of the reference population (19).

A strength of our study is the use of the deuterium dilution method to determine body composition, although 2 limitations of our procedures should be noted: we did not have an objective measure of health before the deuterium dilution sample collection, which is desirable because health status influences body water, and the children were not required to fast because this is difficult for children at this age. Another strength is that we used trained and standardized anthropometrists who were blinded to group assignment and all measurements were done at a central site. Overall, we were able to measure 79% of the children born during the main trial who had not died, with most of the loss to follow-up attributable to relocation of participant households. A potential limitation is the observed differential loss to follow-up between the groups, with a higher loss in the non-LNS group. Despite this differential loss, the groups did not differ in maternal background characteristics (except in asset score which was lower in the LNS group), and adjustment of the statistical models for background characteristics did not change any of our conclusions. In addition, the sample of children included in the follow-up did not differ in maternal background characteristics compared to the full sample of women enrolled in the main trial (except for primiparity), indicating that the results could be generalizable to the study population.

To our knowledge, only 2 previous studies included a follow-up assessment of growth of children exposed to a nutritional supplement during both prenatal and postnatal life, both of which were conducted in the 1970s: the Institute of Nutrition of Central America and Panama (INCAP) longitudinal study in Guatemala (7) and the Bogotá study of Malnutrition, Diarrheal Disease and Child Development in Colombia (8). In both studies, children in the intervention group were taller and weighed more than those in the control group at the end of the supplementation period, as was the case in our study as well. At follow-up, both of the early trials reported sustained effects. Although we did not find a significant overall difference between the non-LNS and LNS groups at 4–6 y, there was a nonsignificant difference of 0.8 cm in height between the IFA and LNS groups, similar to the ∼ 0.6 cm difference between these 2 groups at 18 mo (9), and a significant +1.1 cm difference in height between the non-LNS and LNS groups among children of women who were not overweight. The Bogotá trial showed a difference in height of 2.3 cm at 6 y of age, 3 y after the intervention ended. In the INCAP trial, mean height in the Atole group during adolescence was 1.2 and 2.1 cm greater for males and females, respectively, compared to the Fresco group (the difference was significant only in females). In the earlier trials, the supplementation period extended from pregnancy until at least 3 y of age, whereas supplementation in our trial ended at 18 mo. The types of supplement provided also differed substantially among trials. We provided small-quantity LNS, which provided a similar amount of energy per serving as Atole but less protein. The INCAP trial provided a low-energy fortified supplement to mothers and children in the control group, whereas we provided IFA or MMN to mothers only. The Bogotá trial provided 623 kcal and 30 g protein to each family member and 856 kcal and 38 g protein to the mother daily from week 26 of pregnancy until 36 mo postpartum; the control group received no supplement. The supplements in the Guatemala and Bogotá trials provided more milk than the LNS: at least 20 g of dry milk per serving of Atole in Guatemala (22) and 60 g/d of dry skimmed milk for each household member in Bogotá (8), compared to 4.8 g/d of dry milk from LNS (11). Milk and milk products have been associated with increased linear growth, although the exact mechanism is not known (23). Another difference between these earlier trials and the iLiNS-DYAD Ghana trial is that malnutrition was greater in Guatemala and Colombia at that time, when these lower income countries had a high disease burden, poor access to health care, and poor sanitation, whereas our study population in Ghana had good access to health care and potable water, most of the population had access to improved toilet facilities, and the prevalence of overweight among the mothers at enrollment was high (38.5%). This may explain in part why we saw long-term effects on height only in the group of children born to women who were not overweight. Among children of overweight women, there is likely to be less constraint on the growth of the child because of maternal nutrition (especially in utero), and thus little potential to benefit from LNS, whereas children of nonoverweight mothers may have more potential to benefit from nutritional supplementation as evidenced in a vulnerable Bangladeshi population (24).

Apart from the 2 trials described above, there have been several follow-up studies of food or nutrient supplementation during pregnancy only. A 2015 review and meta-analysis of energy-protein supplementation trials conducted between 1973 and 2014 (25) found no sustained effects of prenatal energy-protein supplementation alone on child growth status at 12 mo of age (26–28) or on height, weight, and body fat at 11–17 y (29).

In another systematic review and meta-analysis of follow-up studies of prenatal micronutrient supplementation trials, the meta-analysis showed no difference between groups receiving prenatal MMNs compared with iron (60 mg) and folic acid in child HAZ or WAZ (17). Two of the trials mentioned, 1 in Burkina Faso (30) and the other in Nepal (31, 32) both reported greater WAZ in the MMN groups at younger ages (1 and 2.5 y), but these differences were not sustained at older ages (2.5 and 8.5 y, respectively). In a separate trial in Nepal (33), there was an increase in mean height in 1 of the intervention groups (vitamin A + iron + folic acid + zinc) compared to the control group (vitamin A alone), but this difference was not evident in the group whose mothers received multiple (>4) micronutrients. The 2 Nepal trials – Janakpur (31, 32) and Sarlahi (33) – and 1 trial in Bangladesh, MINIMat (34), included measurement of body composition. The MINIMat trial showed no difference in skinfold thicknesses, whereas the Sarlahi Nepal trial showed lower skinfold thickness in 1 intervention group (iron + folic acid + zinc) compared to controls at 7.5 y, and the Janakpur Nepal trial showed higher TSF in the MMN group at 2.5 y but not later. There was no reported difference in lean mass or fat mass in either the MINIMat or Janakpur Nepal studies. These findings are consistent with our findings from the follow-up of the iLiNS-DYAD Ghana trial of no differences between the IFA and MMN groups.

We conclude that there was no overall effect of LNS on height or HAZ at 4–6 y of age in this cohort, which had a low stunting rate, but the results suggested an increase in height and HAZ in the LNS group among children of nonoverweight women. Although this finding needs to be confirmed in similar populations, it has potential implications for targeting interventions such as LNS. There was no adverse impact of LNS on child body fatness, suggesting that a small daily quantity of a high-fat fortified supplement in early life does not contribute to child overweight.

## Supplementary Material

nxz005_Supplemental_Files

## Notes

Author disclosures: SMK, SA-A, MA, RRY, TA, RB, MEO, HO, ELP, BMO, and KGD, no conflicts of interest.

Supplemental Tables 1–4 and Supplemental Figure 1 are available from the “Supplementary data” link in the online posting of the article and from the same link in the online table of contents at https://academic.oup.com/jn/.

Abbreviations used: AFA, arm fat area; AMA, arm muscle area; FFM, fat-free mass; FM, fat mass; HAZ, height-for-age *z* score; IFA, iron and folic acid; iLiNS, International Lipid-Based Nutrient Supplements; INCAP, The Institute of Nutrition of Central America and Panama; LNS, lipid-based nutrient supplements; MMN, multiple micronutrients; MUAC, midupper arm circumference; TBW, total body water; TSF, triceps skinfolds; WAZ, weight-for-age *z* score.
