# Supplementary material for: Maternal and Infant Lipid-Based Nutritional Supplementation Increases Height of Ghanaian Children at 4–6 Years Only if the Mother Was Not Overweight Before Conception
Source: J Nutr. 2019 Apr 29;149(5):847–55. doi: 10.1093/jn/nxz005 (PMC6499103; doi:10.1093/jn/nxz005)
Supplement: nxz005_Supplemental_Files [file nxz005_supplemental_files.zip › Online_supporting_material_Table_4.pdf]

Supplemental Table 4: Baseline comparison of intervention groups within the non-overweight subsample of women whose children were in the International Lipid-Based Nutrient Supplements (iLiNS)-DYAD Ghana trial follow-up at 4-6 y[1]

**Maternal Characteristic**	**Non-LNS [*n*=379]**	**LNS [*n*=186]**	***P*-value**
Age (y)	25.7 ± 5.3	25.6 ± 5.2	0.940
Gestational age at enrollment (wk)	16.2 ± 3.2	15.9 ± 3.3	0.275
Years of formal education	7.4 ± 3.3	7.6 ± 3.6	0.421
Married or cohabiting (%)	91.8	91.4	0.864
Asset score[3]	-0.04 ± 0.98	-0.23 ± 0.98	0.032
Nulliparity (%)	39.1	38.7	0.938
Weight (kg)	54.7 ± 5.8	55.3 ± 5.7	0.192
Height (cm)	158.8 ± 5.7	159.4 ± 5.6	0.206
Pre-pregnancy BMI[2] (kg/m^2)	21.7 ± 1.9	21.7 ± 1.8	0.607
MUAC (cm)	25.4 ± 2.3	25.5 ± 2.0	0.658
Triceps skinfold thickness (mm)	14.4 ± 4.6	14.6 ± 4.4	0.678

[1]Values are Mean ± SD unless otherwise stated

[2]Estimated pre-pregnancy BMI was calculated from estimated pre-pregnancy weight (based on polynomial regression with gestational age, gestational age squared, and gestational age cubed as predictors) and height at enrollment

[3]Household asset score was constructed based on ownership of a set of assets (radio, television, refrigerator, and stove), lighting source, drinking water supply, sanitation facilities, and flooring materials, developed into an index (with a mean of zero and standard deviation of one) using principal components analysis.
