# Supplementary material for: Maternal and Infant Lipid-Based Nutritional Supplementation Increases Height of Ghanaian Children at 4–6 Years Only if the Mother Was Not Overweight Before Conception
Source: J Nutr. 2019 Apr 29;149(5):847–55. doi: 10.1093/jn/nxz005 (PMC6499103; doi:10.1093/jn/nxz005)
Supplement: nxz005_Supplemental_Files [file nxz005_supplemental_files.zip › Online_supporting_material_Figure_1.pdf]

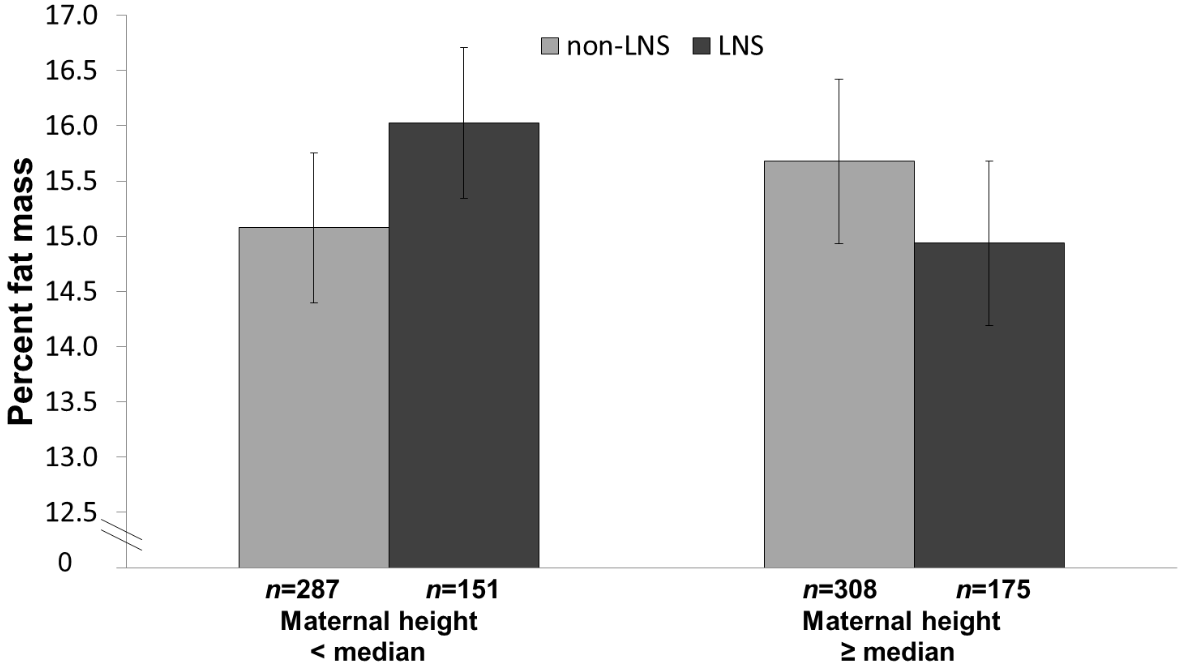

Supplemental Figure 1: Child percent fat mass at 4-6 y by intervention group (LNS vs non-LNS), stratified by maternal height (median = 159.0 cm) at enrollment into the International Lipid-Based Nutrient Supplements (iLiNS)-DYAD Ghana trial. Values represent mean (95% CI) from an ANCOVA model (SAS PROC GLIMMIX). *P*-interaction between maternal height as a continuous variable and intervention group = 0.046, prior to Benjamini/Hochberg correction. LNS = Lipid-based nutrient supplements group; non-LNS = Iron-folic acid group + Multiple micronutrients group
