# Supplementary material for: Maternal and Infant Lipid-Based Nutritional Supplementation Increases Height of Ghanaian Children at 4–6 Years Only if the Mother Was Not Overweight Before Conception
Source: J Nutr. 2019 Apr 29;149(5):847–55. doi: 10.1093/jn/nxz005 (PMC6499103; doi:10.1093/jn/nxz005)
Supplement: nxz005_Supplemental_Files [file nxz005_supplemental_files.zip › Online_supporting_material_Table_1.pdf]

Supplemental Table 1: Background characteristics at enrollment of women whose children were in the International Lipid-Based Nutrient Supplements (iLiNS)-DYAD Ghana trial follow-up at 4-6 y compared to those who were not in the follow-up[1]

Characteristic	In the follow-up [*n*=961]	Not in the follow-up [*n*=358]	*P*-value
**Maternal Characteristics**			
Age (y)	26.8 ± 5.4	26.5 ± 5.7	0.752
Gestational age at enrolment (wk)	16.1 ± 3.3	16.1 ± 3.3	0.180
Years of formal education	7.6 ± 3.4	7.7 ± 3.9	0.534
Married or cohabiting (% [*n*])	93.3 [897/961]	91.1 [326/358]	0.183
Asset score[3]	0.01 ± 0.96	-0.01 ± 1.09	0.576
Primiparous women (% [*n*])	32.2 [309/961]	38.3 [137/358]	0.041
Weight (kg)	62.1 ± 12.07	61.3 ± 11.56	0.226
Height (cm)	159.0 ± 5.7	158.2 ± 5.8	0.300
Pre-pregnancy BMI[2] (kg/m^2)	24.6 ± 4.7	24.4 ± 4.1	0.111
Overweight (BMI ≥25) (% [*n*])	31.8 [300/961]	31.2 [108/358]	0.883
Underweight (BMI <18.5) (% [*n*])	10.9 [103/961]	10.1 [35/358]	0.883
Mid upper arm circumference (MUAC) (cm)	28.0 ± 4.2	27.7 ± 4.0	0.215
Triceps skinfold (mm)	19.0 ± 7.5	19.0 ± 7.4	0.072
**Child Characteristics**			
Sex of child (% boys)	48.0 [447/961]	50.0 [179/358]	0.937

[1]Values are Mean ± SD unless otherwise stated

[2]Estimated pre-pregnancy BMI was calculated from estimated pre-pregnancy weight (based on polynomial regression with gestational age, gestational age squared, and gestational age cubed as predictors) and height at enrollment

[3]Household asset score was constructed based on ownership of a set of assets (radio, television, refrigerator, and stove), lighting source, drinking water supply, sanitation facilities, and flooring materials, developed into an index (with a mean of zero and standard deviation of one) using principal components analysis.
