# Supplementary material for: Maternal and Infant Lipid-Based Nutritional Supplementation Increases Height of Ghanaian Children at 4–6 Years Only if the Mother Was Not Overweight Before Conception
Source: J Nutr. 2019 Apr 29;149(5):847–55. doi: 10.1093/jn/nxz005 (PMC6499103; doi:10.1093/jn/nxz005)
Supplement: nxz005_Supplemental_Files [file nxz005_supplemental_files.zip › Online_supporting_material_Table_2.pdf]

Supplemental Table 2: Anthropometric and body composition measurements of children in the International Lipid-Based Nutrient Supplements (iLiNS)-DYAD Ghana trial at 4-6 y by 3 group (IFA, MMN, LNS)[1]

Variable	IFA [*n*=298]	MMN [*n*=325]	LNS [*n*=338]	*P*-value
Height (cm)	105.9 ± 0.3	106.6 ± 0.3	106.7 ± 0.2	0.075
Height-for-age z-score[1]	-0.65 ± 0.06	-0.50 ± 0.05	-0.49 ± 0.05	0.065
Weight (kg)	16.3 ± 0.1	16.6 ± 0.1	16.7 ± 0.1	0.082
Weight-for-age z-score[1]	-0.80 ± 0.05	-0.67 ± 0.05	-0.67 ± 0.05	0.087
BMI-for-age z-score[1]	-0.60 ± 0.05	-0.55 ± 0.05	-0.55 ± 0.04	0.645
MUAC (cm)	15.3 ± 0.1	15.5 ± 0.1	15.5 ± 0.1	0.087
Triceps skinfold[2] (mm)	6.9 (6.8, 7.1)	7.1 (6.9, 7.2)	7.1 (7.0, 7.2)	0.287
Arm fat area (cm^2)	5.3 (5.2, 5.5)	5.5 (5.3, 5.6)	5.5 (5.3, 5.6)	0.217
Arm muscle area (cm^2)	13.7 (13.5, 13.9)	13.9 (13.6, 14.0)	13.7 (13.6, 13.9)	0.505
Fat mass (FM)[3] (kg)	2.5 ± 0.1	2.6 ± 0.1	2.6 ± 0.1	0.059
% Fat mass[3,4]	15.0 (14.4, 15.5)	15.7 (15.1, 16.2)	15.5 (15.0, 16.0)	0.180
Fat free mass (FFM)[3] (kg)	13.9 ± 0.1	14.1 ± 0.1	14.1 ± 0.1	0.294
% Fat free mass[3]	85.2 (84.6, 85.8)	84.6 (84.0, 85.2)	84.6 (84.0, 85.2)	0.277

[1]Values represent mean and CIs and the difference in mean (95% CI) unless otherwise stated. Results are based on ANCOVA (SAS PROC GLIMMIX). IFA, Iron + Folic Acid tablet; MMN, Multiple Micronutrient tablet; LNS, Lipid-based Nutrient Supplements; MUAC, mid upper arm circumference

[2]Values are geometric means (95% CI) and Ratio of the geometric mean (95% CI). N= 297, 325, 337 (IFA, MMN, LNS).

[3]Values are based on less than the full sample for FM and %FM (291, 308 and 326 for IFA, MMN and LNS respectively) and for FFM, %FFM, (292, 312 and 327 for IFA, MMN and LNS respectively) because some participants did not take part in the body composition procedure, there was insufficient sample for analysis, or there were data collection or lab issues.

[4]The %FM variable was truncated using the value representing the 95th percentile of the distribution to make the distribution more normally distributed.
