# Supplementary material for: Maternal and Infant Lipid-Based Nutritional Supplementation Increases Height of Ghanaian Children at 4–6 Years Only if the Mother Was Not Overweight Before Conception
Source: J Nutr. 2019 Apr 29;149(5):847–55. doi: 10.1093/jn/nxz005 (PMC6499103; doi:10.1093/jn/nxz005)
Supplement: nxz005_Supplemental_Files [file nxz005_supplemental_files.zip › Online_supporting_material_Table_3.pdf]

**Supplemental Table 3:** Prevalence of underweight, stunting, overweight among children in the International Lipid-Based Nutrient Supplements (iLiNS)-DYAD Ghana trial at 4-6 y by 3 group (IFA, MMN, LNS)[1]

| | **IFA [*n*=297]** | **MMN [*n*=325]** | **LNS [*n*=338]** | ***P*-value** |
|---|---|---|---|---|
| **Stunting (HAZ <-2 SD)** | | | | |
| Prevalence (%) | 5.4 | 7.1 | 6.5 | |
| OR (95% CI) | | 1.38 (0.62, 3.03) | 1.19 (0.53, 2.66) | 0.637 |
| **Underweight (WAZ <-2SD)** | | | | |
| Prevalence (%) | 5.7 | 5.5 | 6.2 | |
| OR (95% CI) | | 0.98 (0.43, 2.22) | 1.09 (0.49, 2.41) | 0.940 |
| **Overweight (BMIZ >1 SD)** | | | | |
| Prevalence (%) | 2.4 | 3.4 | 3.0 | |
| OR (95% CI) | | 2.6 (0.65, 10.38) | 2.00 (0.48, 8.33) | 0.271 |
| **Body fat (≥ 20%)** | | | | |
| Prevalence[2] (%) | 12.4 | 16.1 | 17.4 | |
| OR (95% CI) | | 1.28 (0.72, 2.28) | 1.47 (0.84, 2.58) | 0.276 |

[1]Values are the percentage of participants whose response was “yes” for the outcome in question and OR (95% CI) obtained by comparing the groups. Reference = non-LNS group for all outcomes. Results are based on logistic regression (SAS PROC GLIMMIX). IFA, Iron + Folic Acid tablet; MMN, Multiple Micronutrient tablet; LNS, Lipid-based Nutrient Supplements; BMIZ, BMI-for-age z-score; HAZ, height-for-age z-score; WAZ, weight-for-age z-score

[2]Values are based on less than the full sample for overweight [291, 310 and 328 for IFA, MMN and LNS respectively] and some participants did not take part in the body composition procedure, there was insufficient sample for analysis, there were or data collection or lab issues.
